# An Analysis of the Readability of Phacoemulsification Online Resources

**DOI:** 10.7759/cureus.29223

**Published:** 2022-09-16

**Authors:** David F Santos, Gabriel F Santos Malave, Nasir Asif, Natalio Izquierdo

**Affiliations:** 1 Department of Ophthalmology, School of Medicine, Medical Sciences Campus, University of Puerto Rico, San Juan, PRI; 2 Medicine, Icahn School of Medicine at Mount Sinai, New York City, USA; 3 Medicine, Rutgers University, Newark, USA; 4 Department of Surgery, School of Medicine, Medical Sciences Campus, University of Puerto Rico, San Juan, PRI

**Keywords:** medical education, cataract, health literacy, readability, phacoemulsification

## Abstract

Introduction: Cataract is the leading cause of blindness worldwide. Phacoemulsification is now the gold standard for cataract extraction and is greatly needed in low socioeconomic status (SES) communities, rural and older patient populations, and patients with poor vision. This greatly increases the importance of high readability for online resources on this topic. This study aims to assess the readability of online information about phacoemulsification based on readability scores for each resource.

Methods: We conducted a retrospective cross-sectional study. The term “phacoemulsification” was searched online, and each website was categorized by type: academic, physician, non-physician, commercial, social media, and unspecified. The readability scores for each website were calculated using six different readability tests and a composite score that reflects reading grade level was obtained. To evaluate the difference between the categories of websites, analysis of variance (ANOVA) testing was used. All test scores were compared with the 6^th ^grade standard recommendation using a one-sample t-test.

Results: A total of 20 websites were analyzed. Three websites (3/20; 15%) had a score which is correlated with a 6^th^ grade reading level or below. Seventeen websites had a score correlated with a college reading level or above (17/20; 85%). None of the readability scores had a mean below a 6^th^ grade reading level. No category had an average readability score at or below a 6^th^ grade reading level. None of the mean readability scores resulted in a statistically significant difference across categories. All readability tests had an average score which was significantly different from a 6^th^ grade reading level (p<0.001).

Conclusions: This is the first study to focus on the accessibility of online English resources on phacoemulsification and implement multiple standardized readability scores with regards to cataract surgery resources. It provides further overwhelming evidence that online resources on phacoemulsification are too complex for the average patient to understand. Interventions should be implemented to improve readability.

## Introduction

Cataract is the leading cause of blindness worldwide as it accounts for almost 48% of all cases of blindness in over 17 million blind people [1]. A cataract is an opacification of the lens of the eye [2]. It can be unilateral or bilateral. Cataract is most commonly age-related but can be congenital, or secondary to trauma, nutritional, ocular, and systemic disease. Patients will usually experience symptoms of decreased or blurred vision. It occurs gradually and painlessly. It may lead to diplopia or polyopia, colored halos around light, sensitivity to glare, and color vision disturbances [2]. 

While cataract is a disease with a large burden in populations of any socioeconomic status (SES), it leads to more than 90% of the total disability-adjusted life years lost in developing countries [3]. Previous studies [1] have also demonstrated that both the prevalence of cataracts, as well as the severity of symptoms, are worse in rural and low SES populations. 

It is also much more prevalent among older patients since advancing age is the most important risk factor for cataracts [3]. The prevalence of pre-senile cataracts in the 20-39-year age group is 1% versus 88.17% in those over 60 years of age [3]. Therefore, cataract has a large disease burden across all populations worldwide but especially amongst older populations with low income. 

Phacoemulsification is now the gold standard for cataract extraction [4]. This surgery involves an ophthalmic surgeon creating a superior or temporal corneal incision of 2-3 mm and another side port incision at 2-3 o’clock on either side of the main wound. Then after removing the anterior lens capsule (anterior capsulotomy), an ultrasonic probe is used to emulsify lens proteins and aspirate the cataract. This is followed by the implantation of an intraocular lens in the lens bag. Phacoemulsification is done under intravenous sedation, topical or local anesthesia, and the patient can be discharged the same day. Cases without complications usually have improved visual acuity that continues to improve for six weeks [4]. 

The elective decision to undergo cataract surgery depends on the impact of the blurred vision on the patient’s quality of life. Phacoemulsification is not free of complications, including but not limited to posterior capsule opacification, macular edema, retinal detachment, and infections [5]. Achievement of good post-surgical outcomes is dependent on patient adherence to post-operative medications and limited physical activities [6]. Poor compliance can have significant consequences for both the patient and society.

Due to various treatment options, possible complications, and the need for patient compliance, it is vital that patients are well informed. It is common for patients to use online resources as a source of information to make health care decisions [7-9]. Therefore, it is necessary for online information to be accessible. An individual's health literacy is considered the best predictor of their health status [9]. Health literacy is defined as “the degree to which individuals have the capacity to obtain, process, and understand basic health information and services needed to make appropriate health decisions” [10]. However, online health care materials can be difficult for the average American to understand [7]. Over 90 million adult Americans are affected by low literacy [9]. The average American reads at the level of a seventh or eighth-grade student while most online resources are written at the 10th grade level or above [7]. 

The National Institute of Health (NIH), the American Medical Association (AMA), and the United States Department of Health and Human Services (USDHSS) recommend patient education materials be written at or below a 6^th ^grade reading level [7,8,11]. The Joint Commission recommends materials be written at a fifth-grade level or lower [11]. This leads to poor health literacy resulting in increased inpatient hospital service utilization, postoperative complications, and lower patient satisfaction [7,9].

Phacoemulsification is a procedure which is greatly needed in low SES, rural, and older patient populations, and patients with poor vision. This greatly increases the importance of high readability for online resources on this topic. The readability of a text refers to the ease with which a person can understand written material [8]. This study aims to assess the readability of online information about phacoemulsification based on readability scores for each resource. Previous studies [12] have evaluated the readability of online resources on cataract surgery. However, this is the first study to focus on the accessibility of online English resources on phacoemulsification. It is also the first study to implement multiple standardized readability scores with regards to cataract surgery resources.

## Materials and methods

Since this study did not involve patients, Institutional Review Board approval was not required. We conducted a retrospective cross-sectional study design as delineated by McCarthy and co-workers [7]. In April 2022, the term “phacoemulsification” was searched using the search engines Google and Bing. The number of results obtained for each search engine was recorded and is displayed in Table 1. Only the first two pages of hits from each engine were included for analysis. This was due to evidence that demonstrated most people do not look past the second page of search results [13]. Previous readability research has also implemented this cutoff [7]. This further standardizes the readability analysis and makes comparisons easier to achieve. 

**Table 1:** Results by search engine

Search Engine	Hits returned
Google	1,010,000
Bing	359,000

Other exclusion criteria included: duplicate websites, medical journals, websites which only included a video, and websites requiring log in. In accordance with previous studies, medical journals were excluded because they were deemed to be too complex for the average individual [7]. Medical journals are also not always freely accessible. The remaining websites were included in our analysis. 

This was followed by categorizing each website by type. In accordance with a previous readability study, six types were included: academic, physician, non-physician, commercial, social media, and unspecified. Academic refers to any website associated with a university. Physician includes any website owned by a doctor. Non-physician includes websites created by other healthcare providers including pharmacists, physical therapists, radiographers, and occupational therapists. Website ownership was determined through information provided on each individual website. Commercial websites referred to websites which were trying to sell products or contained advertising. Social media includes websites which are linked to social media companies such as Facebook, Instagram, and Twitter. Websites which did not meet any of the five categories were listed as unspecified.

After the websites were categorized, the readability scores for each website were calculated. This was achieved by uploading websites meeting the criteria into WEB FX, an online readability software. WEB FX provides a score for six different readability tests. These are the Flesch-Kincaid Reading Grade Level (FKGL), the Flesch Reading Ease Score (FRES), the Simple Measure of Gobbledygook (SMOG), the Coleman-Liau Index (CLI), the Automated Readability Index (ARI) and the Gunning Fog Index (GFI). WEB FX also provides a reading grade level (RGL) for each website which is a composite score based on the results of the other six scores. For all tests, except FRES, a higher score indicates lower readability since scores correlate with grade level. For the FRES score, a higher score indicates better readability with a score of 80 signifying a 6th grade reading level. A description of each test, the formula used, and an explanation of how to interpret the results is included in Table 2. A description of how to interpret the FRES scale is included in Table 3 [14].

**Table 2:** Descriptions of each readability test used W/S, number words/number sentences; Sl/W, number syllables divided by number of words; CW, complex words (≥3 syllables); L, average number of letters per 100 words; S100, average number of sentences per 100 words; W, number of words [15-17].

Name of Test	Interpretation of results	Description of Test	Formula
Flesch Kincaid Reading Ease	Index Score	A widely used readability formula that estimates the readability of a piece of text. It was developed by Rudolf Flesch in the 1940s. Scores are between 1-100, with a higher score correlating to easier readability.	206.835 − (1.015 ×W/S) − (84.6 × Sl/W)
Flesch Kincaid Grade Level	Grade level	This is a modified version of the Flesch Kincaid Reading Ease that was developed in conjunction with the U.S. Navy. It estimates the U.S grade level to adequately read a piece of text.	(11.8 × Sl/W) + (0.39 × W/S) −15.59
Coleman–Liau index	Grade level	Test developed by Coleman and Liau in 1975. It is based on the principle that measuring readability via the number of letters is a superior measurement over syllable length.	(0.0588L)-(0.296S)-15.8
Simple Measure Of Gobbledygook (SMOG) Index	Grade level	A readability test created by clinical psychologist G. Harry McLaughlin, published in 1969. The SMOG index estimates the years of education the average person needs to comprehend a piece of text. It was found to be the most consistent and practical test when applied to the healthcare setting.	1.0430 × √(CW/S100)+3.1291
Gunning-Fog	Grade level	Designed by Robert Gunning in 1952 to help improve readability for journalism and business writing.	0.4 [(W/S) + 100 (CW/W)]
Automated Readability Index (ARI)	Grade level	Estimates the U.S. grade level needed to read a piece of writing. It is different from the other indexes in that it utilizes character length as opposed to syllable length as per its formula to gauge readability.	4.71 (Ch/W)+0.5 (W/S) – 21.43

**Table 3:** Flesch Reading Ease Score (FRES) scale interpretation

FRES	Reading age
90–100	10–11 years
80–90	11–12 years
70–80	12–13 years
60–70	14–15 years
50–60	16–17 years
30–50	18–20 years
0–30	Graduate

Statistical analysis was done with SPSS software (IBM Corp., Armonk, NY). Significance was set at p-value <0.05%. To evaluate the difference between the six categories of websites, analysis of variance (ANOVA) testing was used. If this achieved significance, post-hoc statistics were calculated. Mean readability scores for each test by category were compared and plotted. All test scores were compared with the 6^th ^grade standard recommendation using a one-sample t-test.

## Results

Twenty-three websites were found on the first two search pages of Bing, and 19 websites were found on Google, for a total of 42 websites. There were 13 duplicate websites across the two search engine searches, meaning 29 unique websites. Nine websites were excluded because they were either medical journals, required logins, or had videos posted. Therefore, a total of 20 websites were analyzed. Table 4 summarizes the websites excluded. Table 5 demonstrates the included websites separated the six categories. As shown in Table 5, the most common category used the physician websites (n=7; 35%) followed by commercial websites (n=5; 25%). 

**Table 4:** Summary of websites excluded

Criteria	Websites (n)
Websites found	42
Duplicates	13
Medical journal	7
Only video	1
Requires login	1
Websites included	20

**Table 5:** Websites by category

Category	Websites (n)
Academic	3
Physician	7
Non-physician	3
Commercial	5
Nonprofit	1
Unspecified	1

The mean values for each readability test are represented in Table 6. None of the readability scores had a mean below a 6^th^ grade reading level. Three websites (3/20; 15%) had a score which is correlated with a 6^th^ grade reading level or below. Eighteen websites had a score correlated with a college reading level or above (18/20; 90%). 

**Table 6:** Readability tests mean values

	FRES	FKGL	GFI	SMOG	ARI	CLI	RGL
Mean +/- SD	42.8 +/- 13	9.2 +/- 1.8	9.7 +/- 2.9	7.7 +/- 1.3	7.8 +/- 1.4	15.4 +/- 3.1	10.3 +/- 1.3

**Figure 1:**
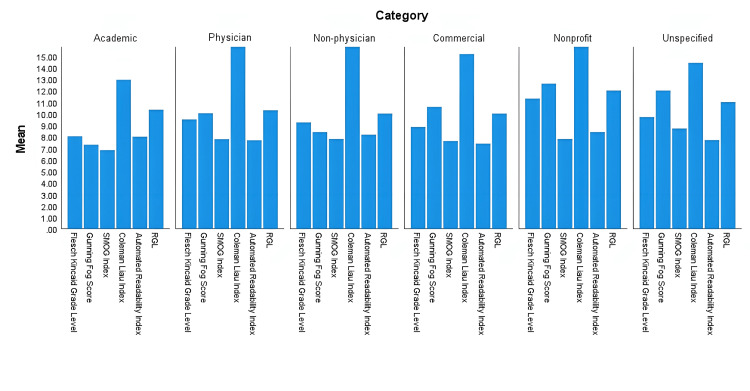
Mean readability scores other than Flesch Reading Ease Score (FRES) across categories

**Figure 2:**
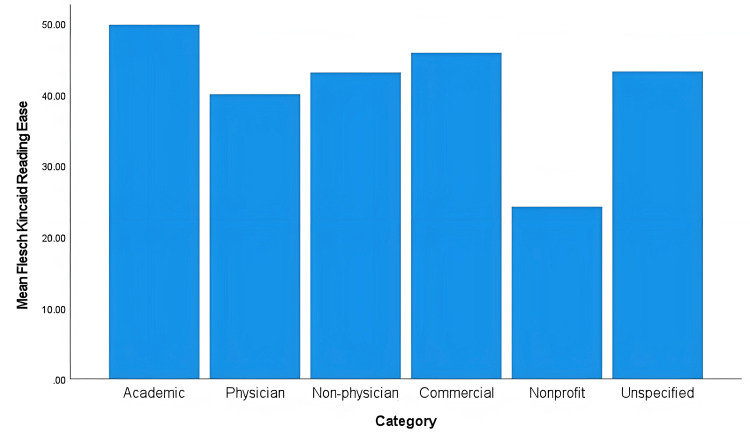
Mean Flesch Reading Ease Score (FRES) scores across all categories

The one-way ANOVA results comparing each test across all website categories are shown in Table 7. The non-profit and unspecified categories were eliminated from the analysis due to their low sample size (n=1). None of the mean readability scores resulted in a statistically significant difference across categories. Figure 1 demonstrates a comparison of mean readability scores other than FRES across all categories. Figure 2 demonstrates this same comparison for the FRES score. No category had an average readability score at or below a 6^th^ grade reading level. 

**Table 7:** One-way analysis of variance (ANOVA) comparison of each readability test across categories FRES: Flesch Reading Ease score; FKGL: Flesch-Kincaid Reading Grade Level; GFI: Gunning Fog Index; SMOG: Simple Measure of Gobbledygook; CLI: Coleman-Liau Index; ARI: Automated Readability Index; RGL: Reading Grade Level.

Type of test	Category (Mean)				p-value
	Academic	Physician	Non-physician	Commercial	
FRES	49.8	40	43.1	45.8	0.75
FKGL	8	9.5	9.2	8.8	0.74
GFI	7.3	10	8.4	10.6	0.79
SMOG	6.8	7.8	7.8	7.6	0.43
CLI	12.9	16	16.2	15.2	0.57
ARI	8	7.7	8.2	7.4	0.92
RGL	10.3	10.3	10	10	0.98

The results of a one-sample t-test comparing the average of each score against the recommended readability score for each test (six for 6^th^ grade reading level) are shown in Table 8. All readability tests had an average score which was significantly different from a 6^th^ grade reading level (p<0.001).

**Table 8:** One-sample t-test comparing readability score with recommended standard SMOG: Simple Measure of Gobbledygook; RGL: Reading Grade Level.

Type of test	Significance	Mean Difference	95% Confidence Interval	
	One-Sided p		Lower	Upper
Flesch Kincaid Reading Ease	< .001	-37.245	-43.3391	-31.1509
Flesch Kincaid Grade Level	< .001	3.17	2.3267	4.0133
Gunning Fog Score	< .001	3.74	2.3709	5.1091
SMOG Index	< .001	1.65	1.0435	2.2565
Coleman Liau Index	< .001	9.385	7.9259	10.8441
Automated Readability Index	< .001	1.77	1.0962	2.4438
RGL	< .001	4.3	3.6907	4.9093

## Discussion

Patients greatly depend on online health care resources as a source of information. It is essential these resources meet the needs of patients by being both informative and easy to understand. This is even more vital with regards to phacoemulsification, a procedure which is commonly indicated in populations with low literacy. 

However, our study demonstrates most websites with information on phacoemulsification do not provide an adequate readability level. For the FRES score, a higher score indicates better readability, with a score above 65 considered an acceptable readability level since it can be interpreted as an 8^th^ or 9^th^ grade reading level. This is around the average American’s reading level of 7^th^ or 8^th^ grade. However, the recommendation for health-related materials is that they be written at a 6^th^ grade level or below; this would correlate to a score of 80 or above. 

For all other tests, a higher score indicates lower readability, since scores correlate with the United States school grade level. Therefore, those tests should all have a score of six or below to have adequate readability for health-related materials. The SMOG test had an average score of 7.7 +/- 1.3. Likewise, the ARI had an average score of 7.8 +/- 1.4. These are around the average American reading level of 7^th^ or 8^th^ grade. However, they are still above the USDHSS 6^th^ grade recommendation. The mean RGL was 10.3 +/- 1.3 which is significantly above the recommended 6^th^ grade level (p<0.001; CI 3.7 to 4.9). All other tests also had a readability score which was significantly above the 6^th^ grade level (p<0.001). This presents a large barrier to patients' understanding of their medical care. Further, this fact impinges on the bioethical principle of autonomy since it limits the ability to provide fully informed consent. 

When compared to previous studies, online resources on phacoemulsification have similar or better readability than publications on liposuction, head and neck surgery, transurethral resection of the prostate (TURP), and prostate artery embolization (PAE) [7,8,18]. Our mean FRES score of 42.8 +/- 13 is significantly lower when compared to a previous study on the readability of cataract surgery websites [12]. The mean RGL of 10.3 +/- 1.3 is comparable to previous findings. Our findings’ similarity with previous readability studies across various disciplines demonstrates this is a common issue in medical care [7,8,12,19,20].

No statistically significant differences in readability were found amongst website categories. This is a crucial finding. It demonstrates patients do not have a range of options with regards to their resources on phacoemulsification. One might expect categories are geared towards certain readers which would lead to significantly different readability scores, such as non-physician and commercial being statistically significantly easier to read than academic or physician websites. This was not the case. An effective intervention would be to provide a variety of resources geared towards varying degrees of readability. In this manner, patients can choose which resource better suits their needs. 

Poor health literacy contributes to more than 73 billion dollars of additional burden to the US healthcare system [9]. The healthcare cost of Medicaid patients with limited literacy is about four times that of those who have adequate health literacy. Low health literacy is associated with poorer health outcomes and poorer use of health care services. Lack of accessible resources for patients with low literacy leads to increased patient costs and harms the patient-physician relationship [9]. Therefore, increasing readability would benefit both the patient and the physician. It would reduce healthcare costs as well as postoperative complications. 

The elderly and those from low SES are likely to have limited health literacy [9]. These are populations which are more likely to need cataract surgery. Therefore, the benefits of improved readability would have the greatest impact on patients likely to need phacoemulsification.

Poor health literacy also partially explains racial disparities in some healthcare outcomes [21]. About 50% of the African American population in the United States reads at or below the 5^th^ grade level [9]. One study found blacks are four times more likely than whites to have an unoperated cataract [22]. Whites were almost 50% more likely than blacks to have undergone cataract extraction before the age of 80. Improving the readability of resources on phacoemulsification could help mitigate the increased prevalence of unoperated cataracts among black patients. 

Limitations to the study include that the access to online resources could not be evaluated. This is a factor which could influence accessibility independent of the readability of the text. Furthermore, only resources in English were evaluated. Due to the large immigrant population in the United States, other languages should also be evaluated. Future studies will elucidate how accessible website searches of phacoemulsification in Spanish are to the Latino population.

## Conclusions

While previous studies have evaluated the readability of phacoemulsification websites, this is the first study to focus on English phacoemulsification websites. It provides further overwhelming evidence that online resources on phacoemulsification are too complex for the average patient to understand. Interventions should be implemented to improve readability and provide varying degrees of complexity. This could help decrease the disease burden of cataracts on the most affected populations. Further studies evaluating Spanish cataract information at online resources are warranted. 

## References

[1] Singh S, Pardhan S, Kulothungan V (2019). The prevalence and risk factors for cataract in rural and urban India. Indian J Ophthalmol.

[2] Nizami AA, Gulani AC (2022). Cataract. StatPearls.

[3] Hashemi H, Pakzad R, Yekta A, Aghamirsalim M, Pakbin M, Ramin S, Khabazkhoob M (2020). Global and regional prevalence of age-related cataract: a comprehensive systematic review and meta-analysis. Eye (Lond).

[4] Gurnani B, Kaur K (2022). Phacoemulsification. StatPearls.

[5] Wensheng L, Wu R, Wang X, Xu M, Sun G, Sun C (2009). Clinical complications of combined phacoemulsification and vitrectomy for eyes with coexisting cataract and vitreoretinal diseases. Eur J Ophthalmol.

[6] Lindstrom RL, Galloway MS, Grzybowski A, Liegner JT (2017). Dropless cataract surgery: an overview. Curr Pharm Des.

[7] Mc Carthy A, Taylor C (2020). SUFE and the internet: are healthcare information websites accessible to parents?. BMJ Paediatr Open.

[8] Sare A, Patel A, Kothari P, Kumar A, Patel N, Shukla PA (2020). Readability assessment of internet-based patient education materials related to treatment options for benign prostatic hyperplasia. Acad Radiol.

[9] Badarudeen S, Sabharwal S (2010). Assessing readability of patient education materials: current role in orthopaedics. Clin Orthop Relat Res.

[10] (2022). National Library of Medicine: current bibliographies in medicine: health literacy. http://www.nlm.nih.gov/archive/20061214/pubs/cbm/hliteracy.html.

[11] Grabeel KL, Russomanno J, Oelschlegel S, Tester E, Heidel RE (2018). Computerized versus hand-scored health literacy tools: a comparison of Simple Measure of Gobbledygook (SMOG) and Flesch-Kincaid in printed patient education materials. J Med Libr Assoc.

[12] Patel AJ, Kloosterboer A, Yannuzzi NA, Venkateswaran N, Sridhar J (2021). Evaluation of the content, quality, and readability of patient accessible online resources regarding cataracts. Semin Ophthalmol.

[13] Kaicker J, Dang W (2016). Assessing the quality and reliability of health information on ERCP using the DISCERN instrument. Health Care Curr Rev.

[14] Hartley J (2016). Is time up for the Flesch measure of reading ease?. Scientometrics.

[15] Coleman M, Liau L (1975). A computer readability formula designed for machine scoring. J Appl Psychol.

[16] Kincaid JP, Fishburne RP Jr, Robert P, Richard L, Brad S (1975). Derivation of new readability formulas (automated readability index, fog count and Flesch reading ease formula) for navy enlisted personnel. DTIC.

[17] Dubay WH (2004). The Principles of Readability. http://chrome-extension://efaidnbmnnnibpcajpcglclefindmkaj/https://files.eric.ed.gov/fulltext/ED490073.pdf.

[18] Vargas CR, Ricci JA, Chuang DJ, Lee BT (2016). Online patient resources for liposuction: a comparative analysis of readability. Ann Plast Surg.

[19] Schmitt PJ, Prestigiacomo CJ (2013). Readability of neurosurgery-related patient education materials provided by the American Association of Neurological Surgeons and the National Library of Medicine and National Institutes of Health. World Neurosurg.

[20] Wong K, Levi JR (2017). Readability trends of online information by the American Academy of Otolaryngology-Head and Neck Surgery Foundation. Otolaryngol Head Neck Surg.

[21] Berkman ND, Sheridan SL, Donahue KE, Halpern DJ, Crotty K (2011). Low health literacy and health outcomes: an updated systematic review. Ann Intern Med.

[22] Sommer A, Tielsch JM, Katz J (1991). Racial differences in the cause-specific prevalence of blindness in east Baltimore. N Engl J Med.

